# Instruments and scales for assessing schizo-obsessive disorder: a systematic review

**DOI:** 10.1192/j.eurpsy.2025.1710

**Published:** 2025-08-26

**Authors:** Y. A. Ferrao, J. V. B. Ferrão, P. C. Miranda, A. P. Feier, G. M. Ferreira, R. Massuda, D. Fragoso, M. S. Barbosa

**Affiliations:** 1 Psychiatry, UFCSPA; 2 Psychiatry, HMIPV; 3 Medicine, PUCRS, Porto Alegre; 4 Psychiatry, UFPR, Curitiba, Brazil

## Abstract

**Introduction:**

Recent evidence suggest the nosological entity called Schizo-Obsessive Disorder (SchizoOCD), similar to Schizoaffective Disorder. Some authors argued that obsessions and delusions would be on a continuum, which justify the difficulty in distinguishing obsessive from delusional thoughts, and compulsions from stereotypical behaviors. In order to assist in the screening, monitoring or treatment of such disorders, instruments as scales and questionnaires may be important tools in psychiatric practice.

**Objectives:**

This systematic review investigated the most frequent instrumentsused to assess SchizoOCD.

**Methods:**

We systematically reviewed articles up to 2015 in English, Portuguese and Spanish at PubMed, Scielo and Embase databases. We included studies with humans, no age limitation, with OCS or diagnosis of OCD and schizophrenia or psychotic symptoms. Systematic review articles, meta-analysis, letters to the editor and case reports were excluded, as well as articles that did not use assessment instruments for the diagnosis of schizophrenia comorbid with OCD. The methodological and clinical data extracted from the articles are described at the results.

**Results:**

A total of 9,833 articles were selected, but 53 were read. Cross-sectional studies were the most frequent (n=39; 73.6%), followed by cohort studies (n=9; 17.0%).The total sample size of Schizo-OCD patients was 2,605 patients (in 44 studies), of which 44.7% (n=1,164) were female. The mean age and the age of onset of the disorders are described in Table 1. Only 23 (44.4%) of the studies described the psychiatric comorbidities (2 (3.8%) studies reported that the patients had no comorbidities). The most frequent comorbidities were Major Depression (n=18; 34%) and Substance Use Disorder (n=9; 17.0%). The used diagnostic instruments or interviews are listed in Table 2. Table 3 describes the scales used to assess the severity of Schizophrenia and/or OCD symptoms. From a psychopathological point of view, only 9 (17.0%) of the articles described psychotic symptoms in more detail. For OCD, 15 (28.3%) of the articles detailed the obsessive-compulsive symptoms.

**Image:**

**Figure:**
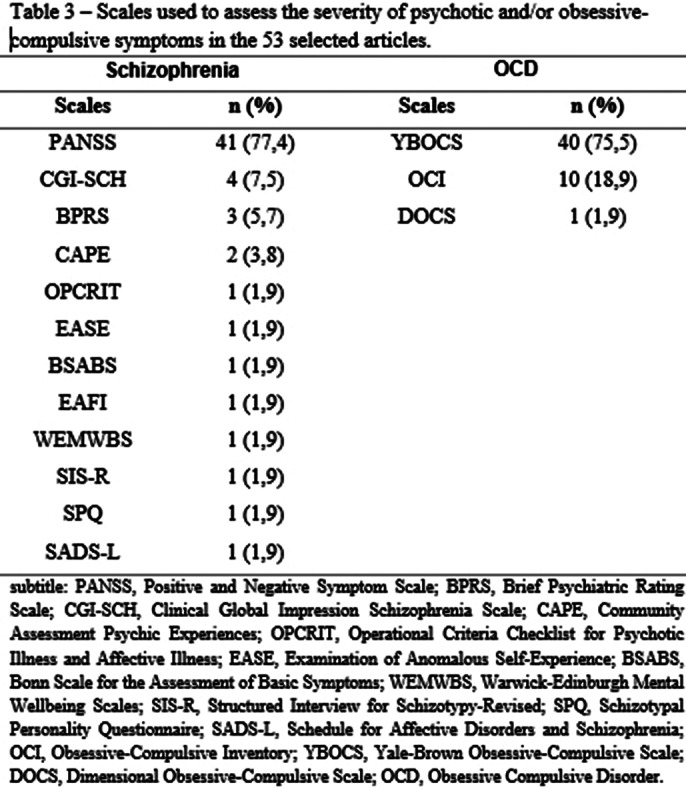

**Image 2:**

**Figure:**
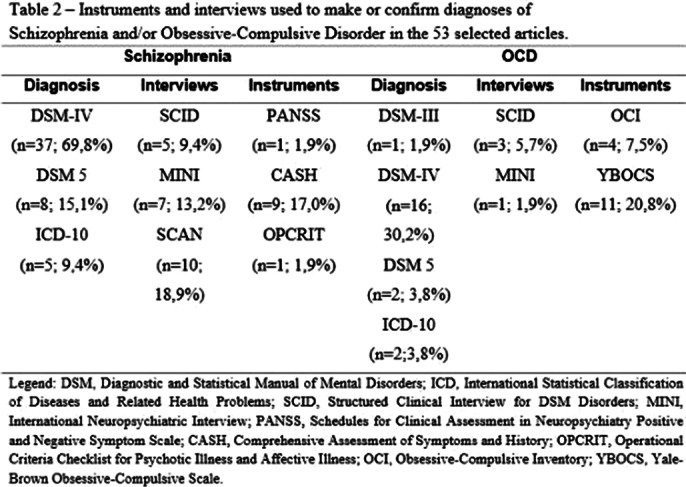

**Image 3:**

**Figure:**
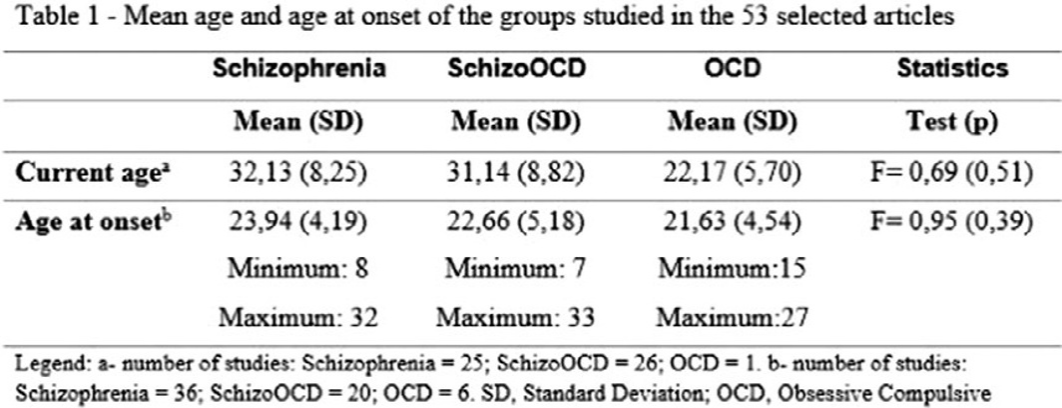

**Conclusions:**

Few studies in the literature used scales to discriminate psychotic and obsessive-compulsive aspects in patients with the alleged diagnosis of Schizo-OCD. Scales for measuring symptom severity such as PANSS and YBOCS were widely used in the studies, indicating that their application in clinical practice can serve as an aid during treatment management. Specific scales and instruments for Schizo-OCD were not found and we suggest as a future perspective the development of a new tool to assess symptoms and to elucidate possible symptomatic confusions.

**Disclosure of Interest:**

None Declared

